# Evidence for Long-Term Impact of *Pasos Adelante*: Using a Community-Wide Survey to Evaluate Chronic Disease Risk Modification in Prior Program Participants

**DOI:** 10.3390/ijerph10104701

**Published:** 2013-10-01

**Authors:** Scott C. Carvajal, Noelle Miesfeld, Jean Chang, Kerstin M. Reinschmidt, Jill Guernsey de Zapien, Maria L. Fernandez, Cecilia Rosales, Lisa K. Staten

**Affiliations:** 1 Arizona Prevention Research Center, Mel and Enid Zuckerman College of Public Health, University of Arizona, 1295 N. Martin Ave, Tucson, AZ 85724, USA; E-Mails: noellem@email.arizona.edu (N.M.); jean0131@email.arizona.edu (J.C.); kerstin@email.arizona.edu (K.M.R.); dezapien@email.arizona.edu (J.G.Z.); chachaml@hotmail.com (M.L.F.); crosales@email.arizona.edu (C.R.); 2 Richard M. Fairbanks School of Public Health, Department of Social and Behavioral Sciences, Indiana University, 714 N. Senate Avenue, EF 250, Indianapolis, IN 46202, USA; E-Mail: lkstaten@iupui.edu

**Keywords:** chronic disease, prevention and control, health behavior, diabetes prevention and control, Hispanic health, community health services, program evaluation

## Abstract

Effective community-level chronic disease prevention is critical to population health within developed and developing nations. *Pasos Adelante* is a preventive intervention that aims to reduce chronic disease risk with evidence of effectiveness in US-Mexico residing, Mexican origin, participants. This intervention and related ones also implemented with community health workers have been shown to improve clinical, behavioral and quality of life indicators; though most evidence is from shorter-term evaluations and/or lack comparison groups. The current study examines the impact of this program using secondary data collected in the community 3–6 years after all participants completed the program. A proportional household survey (N = 708) was used that included 48 respondents who indicated they had participated in *Pasos*. Using propensity score matching to account for differences in program participants *versus* other community residents (the program targeted those with diabetes and associated risk factors), 148 natural controls were identified for 37 matched *Pasos* participants. Testing a range of behavioral and clinical indicators of chronic disease risk, logistic regression models accounting for selection bias showed two significant findings; *Pasos* participants were more physically active and drank less whole milk. These findings add to the evidence of the effectiveness of *Pasos Adalente* and related interventions in reducing chronic disease risk in Mexican-origin populations, and illustrate the use of innovative techniques for using secondary, community-level data to complement prior evaluation research.

## 1. Introduction

The implementation of effective health promotion programs that target chronic disease prevention and control is crucial to addressing population health as well as associated economic and psychosocial burdens. The total cost of diabetes alone has been estimated at $174 billion annually [1]. One of the key challenges to this effort is the ability to document long-term, sustained health and behavior improvements that can be attributed to specific programs or interventions. While there are a number of studies that have documented long-term changes in predominantly non-Hispanic white participants [2] and others have included US Hispanics in their sampling [3], few population health studies have examined the long-term effects of interventions exclusively targeting Mexican origin Hispanics. The need for effective and culturally appropriate programs is of particular importance due to the growing Mexican origin Hispanic population and their increasing rates of diabetes and related endocrine disorders [4,5].

Diabetes prevalence in the US for the Hispanic population over the age of 20 is estimated at 11.8 %, compared to 8.3 % prevalence for the general population [1]. Mexican-Americans (13.3%) have the second highest rate of diabetes within the Hispanic population, slightly lower than Puerto-Ricans (13.8%) [1]. The rate for Mexican-descent persons living along the US-Mexico border is even higher compared to Mexican-descent persons and Hispanics living elsewhere in the US [6,7]. Data from a 2001–2002 diabetes prevalence study indicated that 15.7% of the US-Mexico border population has diabetes [6]; other studies also show that rates and complications are further escalated in low socioeconomic status, rural, and US-Mexico border areas [8,9]. Obesity and being overweight, both highly prevalent in the border region, are also risk factors for developing Type 2 diabetes [6]. In 2002, there were 1.5 million individuals living on the US side of the US-Mexico border that were overweight and obese [6]. These individuals have a 2.8 times greater risk of developing diabetes compared to those with normal weight along the border [6]. The burden of diabetes is compounded by serious, costly complications, which include heart disease, stroke, hypertension, blindness, kidney disease, and amputations [1] as well as other negative impacts on quality of life [10,11].

The *Pasos Adelante* program was developed to target the prevention and control of cardiovascular disease, diabetes, and obesity in the Mexican-origin population and was initially implemented along the Arizona, US-Sonora, Mexico border. *Pasos* is based on the National Heart, Lung, and Blood Institute’s *Su Corazón, Su Vida*/*Your Heart, Your Life* [12] curriculum and is a theory-based 12-week community health worker(also known as *promotores* in Hispanic communities)-led intervention. The *Pasos* curriculum includes a focus on physical activity, nutrition, emotional and mental health education, and community advocacy. As a core element, the program facilitates regular social walking groups [13,14,15]. Initial evaluation of the program demonstrated that program participants showed significant improvements in self-reported post-test assessments in amount of moderate to vigorous walking per week and the number of participants walking after the 12-week intervention [14]. Participants also significantly increased consumption of fruits and vegetables and decreased consumption of sweetened sodas and sweetened hot drinks [14]. Subsequent evaluations in a different US-Mexico border community documented that participation in *Pasos* resulted in significant improvements in body mass index, health-related quality of life, depression, blood pressure, glucose levels, and total cholesterol for Hispanic Mexican-American participants aged 40 years and older [13,15]. These improvements were observed directly after completing the program (three months after the program began) and were maintained at another assessment three months after program completion [13,15].

*Pasos Adelante* has been recognized by the Centers for Disease Control and Prevention (CDC) to be effective at improving lifestyle behaviors [16]. The CDC in 2012 identified *Pasos* as a research-tested intervention that addresses one of the ten CDC “winnable battles” and quickly achieved measurable impact in the targeted areas of nutrition, physical activity, and obesity [16]. This determination was based on reviews of the evidence that participation in *Pasos,* as well as with similar programs evaluated with randomized controlled trials [17,18], lowers chronic disease risk in Hispanic adults immediately post program completion through assessment up to six months past baseline. However evidence for longer term impact of these programs is currently lacking, as is evidence specific to *Pasos* with comparisons to controls. The current study utilizes a secondary analysis from a random household survey in the community where the major trial of *Pasos Adelante* was implemented to explore long-term behavior change patterns and biological markers in a subset of respondents who indicated they had participated in that program.

## 2. Methods

The current study utilized data from the Douglas Community Health Survey (2010–2011). These data were used to examine the long-term effects of participation in *Pasos Adelante* in three ways. First, the behavioral and clinical outcomes of *Pasos* participants were compared to all other community members in the survey. This initial examination is particularly susceptible to selection bias, as participants were more likely to be recruited into *Pasos* if they had diabetes or related chronic disease risk factors. Next, two methods, subsequently described, that optimally account for selection biases given our data considerations were employed. Of note, the duration between the completion of all *Pasos Adalante* intervention elements and the implementation of the Douglas Community Health Survey ranged from a minimum of three years to a potential maximum of six years.

### 2.1. Design/Participants and Sampling

The Douglas Community Health Survey was a random proportional community-wide survey of 708 residents conducted between October 2010 and September 2011. The primary purpose of this survey was to re-assess community health and social needs. This re-assessment followed extensive community and academic partnership efforts to reduce diabetes, other chronic disease burdens, and access to health care barriers over a 12-year period; efforts in part spawned from a related 1997–1998 health and needs assessment survey in this community [19]. *Pasos Adelante* participants were not specifically targeted for inclusion in the community-wide survey nor was the sampling powered for interpretations about this program.

The Douglas Community Health Survey was representative of the Arizona, US-Sonora, Mexico border community municipality of Douglas and its surrounding area. This is a rural and health services underserved community [20,21]—and the largest target area of *Pasos* intervention research projects [14,15]. The random household sampling was based on a total of 17,387 residents from the municipality of Douglas [22] and 1,744 residents from the adjacent unincorporated area of Pirtleville [22] that shares common services. Strata of census blocks were identified by ethnicity (Hispanic/non-Hispanic; census data show approximately 99% of the Hispanic residents in this community are Mexican origin [22]) and socioeconomic status (SES). Occupied housing units were randomly chosen from the selected census area. The survey was conducted by trained bilingual community health workers from the community who visited each household to explain the purpose of the study and to collect informed consent. All residents over age 18 were invited to participate in this study with no monetary compensation. The study yielded a total of 708 respondents and corresponded to an 86% participation rate—a testament to the rapport of the community health workers who implemented the survey and the interest of community members in their own health assessment. All objectives, materials, and protocols for the study were reviewed and approved by the University of Arizona’s Institutional Review Board. 

### 2.2. Adjusting for Selection Bias

Two methods were used to address selection bias, which is a chief threat to causal interpretation in non-randomized program evaluation designs. This was done by adjusting for differences in both *Pasos* and non-*Pasos* participants, aside from their program participation. The first method adjusts for selection bias through a multivariable model risk adjustment [23]. This is a versatile technique and the most common method of controlling for selection bias with measured confounders [24]. The second method adjusted for selection bias using propensity-score matching [23,25,26]. This method can determine a natural control group and may improve the causal inferences afforded from non-randomized designs [27], thus for our study controls were respondents from the same community who are empirically most similar to *Pasos* participants with the exception of participation in the program. Propensity score matching was selected as it has some advantages over related methods (e.g., multivariable adjustment, stratification, other techniques using propensity scores). Specifically, it is both relatively robust to covariate imbalances and is a method where confounds are adjusted for prior to conducting analysis with the outcome variables [24,26].

### 2.3. Procedure/Measures

The survey included objectively measured clinical indicators. These include a finger stick blood collection for glucose testing and anthropometric measures (height, weight, waist, and hip). The main component of the survey was a self-report questionnaire, which included documentation on behavioral risk factors and protective factors (e.g., activity, diet, diabetes-related beliefs, health history, access to care), and community-based program exposure. Assessment methods of the behavioral factors are summarized in Table 1.

**Table 1:** Assessment methods of program exposure and behavioral outcomes.

*Behavioral Variables*	*Assessment Methods*
Program Exposure	“Yes” to “Have you participated in the *Pasos Adelante* program?”
Diabetes-Related Control Beliefs	“Yes” to 2 questions: “Do you think that diabetes can be prevented?” & “If someone develops diabetes, do you think it can be controlled?”
Activity Level	International Physical Activity Questionnaire (IPAQ; Short Form) [28]. Sedentary behavior: sitting 5 h or more/day [29]. Physical activity: walking 150 min or more/week and whether they met CDC recommended level of exercise (150 min of moderate intensity exercise/week or 75 min of vigorous intensity exercise/wk) [30].
Fruit and Vegetable Consumption	Behavioral Risk Factor Surveillance System (BRFSS) [31,32]. Whether respondents ate three or more servings of fruit and vegetables. (Note only 3% ate five or more a day)
Fatty Milk Consumption	Determined by whether participants answered that they drank “whole milk.” [33]
High Sugared Drink Consumption	Determined by whether participants drank on average >1 drink a day from soda or other sugared drink (lemonade, iced tea, kool-aide, horchata, Gatorade) [33]

Clinical outcomes for this study included blood glucose and two objective indicators of obesity. Blood glucose samples were collected using capillary draw with the OneTouch system—a well performing device in validation studies [34]. If participants did not have anything to eat eight hours prior to the draw, the sample was considered fasting. If participants had eaten within eight hours, the sample was considered postprandial. Elevated glucose was defined by having a fasting blood glucose level that is greater than or equal to 126 mg/dL or postprandial glucose that is greater than or equal to 200 mg/dL [35]. Body mass index (BMI) was calculated by using weight (kg)/(height (m))^2^ derived from medical grade scales and stadiometers. Elevated Waist-to-hip Ratio (WHR) was categorized by rates >0.99 for men and >0.92 for women, suggested optimal criteria for elevated chronic disease risk within our predominantly Mexican-descent Hispanic sample [36].

Selected covariates and potential confounding variables included demographic and acculturation markers known to be associated with diabetes status and co-morbid conditions in various US Hispanic populations [37,38,39,40,41,42]. Hispanic ethnicity was defined by a yes to the following question: “Do you consider yourself to be of Hispanic origin or descent (such as Mexican, Mexican-American, Latino/a, Latin American or Chicano/a)? (¿Se considera de origin Hispano(a), (Mexicano(a) Mexico-Americano(a), Latino(a), Latino Americano(a), Chicano(a)?)”. The demographic variables for the current analysis included age, gender, Hispanic (we estimate 99% are of Mexican origin from our nation of origin data and census data), marital status, years of education, and having private health insurance, Medicaid, and/or Medicare. Acculturation markers included Spanish and English proficiency derived from a well-tested scale [43], being born in the US, and number of years living in the US. Other potential confounders—variables that could relate to whether the survey respondents participated in the intervention program—included years residing in the community, whether they had been told they had diagnosed diabetes seven or more years before they completed the current survey (before the *Pasos* implementation began in this community [14,15]), and a question on beliefs toward community empowerment, an indicator of an individual’s perception of social capital.

### 2.4. Analysis of the Effects of Pasos Adelante

The initial analysis was a test of unadjusted differences between participants who reported *Pasos* participation (n = 48) and those who did not (n = 660), executed with bivariate logistic regression analysis. Next, multivariable logistic regression analyses were used to generate an odds ratio for program participation relating to each outcome variable statistically controlling for all potential covariates and confounders.

The next phase of the analysis identified a natural control group from the remaining 660 respondents and their respective matches from the pool of *Pasos* participants (n = 48), and bivariate logistic regression models were conducted with these propensity score matched samples [24]. These models reflect the difference in odds of each outcome attributable to those who participated in *Pasos* compared to their natural controls matched from all other survey respondents.

The initial stage of propensity score matching required a multiple logistic regression model predicting *Pasos* participation in the full survey (N = 708). This analysis estimates the likelihood that one of the persons in the survey would indicate they participated in *Pasos* (n = 48) given their responses in all potential covariates and confounder variables previously described—thus the predicted probability of being in *Pasos* from this multivariable logistic model becomes the propensity score. Given our much larger numbers of potential controls and relatively few *Pasos* participants (<50) four control cases were selected in order to balance statistical power while identifying the most closely matched controls. A well performing greedy matching approach was used [24], where the selection of potential matches of each *Pasos* participant was executed randomly until all such matches to each participant were exhausted.

For the *Pasos* participant (and the four matched controls) to be included there had to be two controls with a higher adjacent propensity score (nearest neighbor matching [24,25]) and two with a lower adjacent propensity score, and both within a specified caliper distance. The latter in our case was 0.20 of the standard deviation of the logit of the propensity score; such a criterion is expected to eliminate 98% of the bias in a crude estimator of differences in proportion [25].

Using the above criteria, the matched samples included 37 *Pasos* participants and their 148 counterparts from the survey, the latter respondents represent what we term a natural control group. There were no significant differences between the matched groups in all 15 covariates and potential confounding variables examined; this supports the appropriateness of using these propensity score matched groups in the outcome phase of this analysis. Given the modest sample size and the lack of systematic differences in covariates [24], bivariate logistic regression was then used to test for differences in these groups in the proportion reflected in each outcome variable. 

## 3. Results

Behavioral factors were first analyzed descriptively in the total survey sample (N = 708). Inactivity, sitting 5 or more hours per day, was reported by 11% of the respondents. Physical activity, categorized as 150 min or more of walking per week, was reported by 39% of respondents, and meeting CDC recommended exercise criteria, categorized as 150 min of moderate intensity exercise per week or 75 min of vigorous intensity exercise per week, was reported by 59% of respondents. Only 3% of the population reported eating five or more servings of fruits and vegetables per day and 32% reported eating three or more servings of fruits and vegetables per day. Fatty (whole) milk consumption was reported by 23% of the population and high consumption of sugared drinks was reported by 64% of the sample. Analysis of clinical results revealed that 8% of all respondents had elevated glucose. A calculated BMI of 30 or above, a standard BMI level for determining obesity, was present in 41% of the sample. Lastly, elevated Waist-to-Hip ratio (WHR) was found in 27% of the sample. 

For further descriptive analyses, the 708 respondents were divided into three sample groups: those who participated in *Pasos* program (n = 48), natural controls (n = 148), and propensity score matched *Pasos* participants (n = 37). Table 2 also presents demographic and descriptive characteristics of these groups. Mean age was higher in *Pasos* participants (59.1 ± 14.2) than total sample (52.8 ± 19.0); and majority of the participants were females in all groups. Also, the *Pasos* participants group, natural controls, and *Pasos* matched participant group were all of Hispanic (Mexican) origin, compared to the total study sample of 92%. Over half of the participants (59.6%) reported to be married (or in a consensual union). The years of education received were around 11 years for all groups. In the total sample there were greater number of Medicaid beneficiaries (40.8%) compared to Medicare (28.2%) and private insurance (25.8%). This pattern of having greater participants enrolled in Medicaid than Medicare or private insurance was also true for the *Pasos* participant group, natural controls group, and *Pasos* matched participants group. Regarding country of origin, total population indicated that 43.2% were born in US, whereas the percent of US born was 19.1% for all *Pasos* participants. The total population also indicated the longest US residency of 35.9 ± 21.6 years, compared to the remaining three groups that had residency of approximately 17 years. Residency in Douglas has a slightly different pattern. All *Pasos* participants had lived in Douglas (29.6 ± 22.6) more than the total population (28.7 ± 20.6). English Language Score was slightly higher in the total population (5.7 ± 2.7) than all three groups (Spanish Language Score was not estimated because of the high ceiling effects for the items, all substantive variance in our acculturation measure was thus on the English dimension).

In reporting whether participants had diabetes diagnosed seven years prior to the survey, all *Pasos* participants had greater percent of prior diabetes diagnosis (27.7%) than the total population (13.4%); the propensity score matched groups were in between those estimates. The belief of community social capital was highest in total population (67.1%) than all *Pasos* participants (61.7%), natural controls (66%), and *Pasos* matched participants (62.2%). 

Diabetes control-related beliefs were highest in *Pasos* matched participants (78.4%), followed by in all *Pasos* participants (76.6%), natural controls (70.0%), and total population (65.7%). For whether participants met CDC recommended level of exercise, *Pasos* matched participants demonstrated the highest physical activity (81.1%) relative to all other groups. Participants’ level of inactivity, measured by hours of sitting, was lowest among *Pasos* matched participants (8.1%). Approximately 43% of all *Pasos* participants reported that they walked more than 150 min or more per week. Among the all *Pasos* participants group, 34% (the highest percentage compared to other groups reported) consumed at least 3 servings of fruits and vegetables per day. Fatty milk consumption was highest in total sample (23.3%) and lowest in *Pasos* matched participants (8.1%). More than half of the total sample (64%) frequently consumed sugared drinks compared to approximately half of all *Pasos* participants (51.1%).

**Table 2:** Descriptive characteristics and frequencies of the study variables in the full sample, *Pasos Adelante* participants, and the matched sub-sample groups.

*Variable*	Total sample; N = 708	All *Pasos* participants; n = 48	Natural controls; n = 148	*Pasos* matched participants;n = 37 *
Age (mean ± SD)	52.8 ± 19.0	59.1 ± 14.2	57.3 ± 18.2	57.0 ± 13.2
Gender Male Female	37.3% 62.7%	10.6% 89.4%	10.9% 89.1%	13.5% 86.5%
Hispanic (Mexican) Origin (Yes)	91.7%	100%	100%	100%
Marital Status(Yes)	59.6%	59.6%	52.4%	56.8%
Education in years (mean ± SD)	11.4 ± 2.6	11.5 ± 2.5	11.2 ± 2.8	11.2 ± 2.5
Insurance Status Medicaid Medicare Private	40.8% 28.2% 25.8%	38.3% 34.0% 21.3%	36.7% 35.4% 25.9%	43.2% 29.7% 21.6%
US born (Yes)	43.2%	19.1%	21.8%	21.6%
Years in the US (Mean ± SD)	35.9 ± 21.6	17.1 ± 5.1	17.2 ± 5.6	17.3 ± 4.8
Years in Douglas (Mean ± SD)	28.7 ± 20.6	29.6 ± 22.6	28.6 ± 20.3	27.7 ± 20.3
English Language Score(score of 2 to 8; mean ± SD)	5.7 ± 2.7	4.8 ± 2.4	4.7 ± 2.7	4.8 ± 2.4
Had diabetes diagnosed ≥7 years ago	13.4%	27.7%	19.7%	21.6%
(High) Community empowerment beliefs	67.1%	61.7%	66.0%	62.2%
(High) Diabetes control-related beliefs	65.7%	76.6%	70.0%	78.4%
Meet CDC recommended Physical Activity per week	59.3%	76.6%	60.5%	81.1%
Sit ≥5 h/day	11.0%	10.6%	10.3%	8.1%
Walk ≥150 min/day	38.6%	42.6%	35.0%	40.5%
Consume ≥3 fruits/vegetables per day	32.7%	34.0%	32.0%	29.7%
Consume fatty milk	23.3%	8.5%	23.1%	8.1%
Consume >1 sugared drink per day	64.0%	51.1%	63.3%	54.1%
Elevated Glucose	8.1%	10.6%	11.6%	5.4%
BMI ≥30	40.1%	51.0%	34.7%	51.4%
Elevated WHR	26.9%	26.1%	27.4%	27.8%

* Subset of the 48 participants.

The percent of elevated glucose was lowest in *Pasos* matched participants (5.4%). Among respondents with a BMI of 30 or greater, *Pasos* matched participants (51.4%) had the largest percentage of respondents, followed by all *Pasos* participants (51%), total population (40.1%), and natural controls (34.7%). Another obesity measure, Waist-to-Hip Ratio (WHR), was examined; *Pasos* participants had slightly lower levels of elevated WHR (>0.99 for men and >0.92 for women) than other sample groups.

**Table 3:** Crude and adjusted odds ratios for *Pasos* participation impact on study outcomes.

Variable	COR		AOR ^a^		COR ^b^	
	COR	95%CI; n	AOR	95%CI; n	COR	95%CI; n
Diabetes beliefs	1.78	0.89–3.56; 706	1.89	0.91–3.91; 706	1.55	0.66–3.65; 184
Sit ≥5 h/day	0.95	0.365–2.48; 704	1.03	0.37–2.89; 704	0.77	0.21–2.82; 183
Walk ≥150 min/week	1.19	0.653–2.16; 706	1.49	0.79–2.81; 706	1.28	0.61–2.69; 184
**Meet rec. physical activity/week **	2.36 *	**1.18–4.72; 706**	2.82 ***	**1.33–5.97; 706**	2.79 *	**1.03–5.60; 184**
Consume ≥three fruits and vegetables per day	1.07	0.57–2.00; 704	1.25	0.65–2.40; 704	0.90	0.41–1.98; 184
**Consume fatty milk**	0.29 *	**0.10–0.82; 706**	0.34 *	**0.12–1.00; 706**	**0.29**	**0.09–1.02; 184**
Consume >1 sugared drink/day	0.56	0.31–1.02; 706	0.76	0.40–1.46; 706	0.73	0.68–1.42; 184
Elevated Glucose	1.39	0.53–3.66; 706	0.76	0.25–2.35	0.44	0.10–1.98; 184
BMI ≥30	1.54	0.85–2.79; 706	1.71	0.91–3.21; 706	1.99	0.96–4.12; 182
Elevated WHR	0.96	0.49–1.90; 698	0.89	0.43–1.86; 698	1.02	0.45–2.30; 182

Note: COR = Crude Odds Ratio. AOR=Adjusted Odds Ratio. CI = Confidence Interval. n = sample size for that analysis. (a) Adjusted for age, gender, Hispanic/Mexican origin, marital status, education, insurance, country of origin, residency, English language score, diabetes diagnosis >7 years prior, and beliefs of community empowerment. (b) crude odds estimated with matched propensity score cases based on the covariates in the multivariable model. *** *p* ≤ 0.001; ** *p* ≤ 0.01; * *p* ≤ 0.05.

Table 3 describes crude and adjusted odds ratios of behavioral and clinical outcomes associated with *Pasos Adelante* participation. All estimates for meeting CDC recommended levels of physical activity were significant, and higher odds for *Pasos* participants were observed by a factor over two in all models. In the unadjusted model with the full sample, the COR was 2.36 (95% CI = 1.18–4.72). In the multivariable adjusted model, the AOR was higher at 2.82 (95% CI = 1.33–5.97). In the propensity score matched groups, the COR was 2.79 (95% CI = 1.03–5.60). Other significant differences were observed in the models with fatty milk consumption. The COR was 0.29 (95% CI = 0.10–0.82) and the AOR was 0.34 (95% CI = 0.12–1.00); for both models those in *Pasos* were about one third as likely to drink whole milk as those who did not participate in *Pasos*. Whole milk consumption was not significant in the matched propensity score cases though the estimate was similar to that from other models (COR = 0.29; P = 0.06). No other models showed participation in *Pasos* was significantly associated positively or negatively with the behavioral or clinical outcomes.

## 4. Discussion

The current study examined the impact of *Pasos Adelante* on a range of behavioral and clinical indicators of chronic disease risk measured 3–6 years after the initial project was implemented. This study was executed using a secondary analysis of a community representative survey of adults designed and implemented to provide a health needs assessment for that community. The analysis employed two strategies to reduce the influence of selection bias, attributable due to the fact that *Pasos* participants were likely to have diabetes or associated chronic disease risk factors that influenced the likelihood of program enrollment. These techniques were regression-based statistical adjustment and propensity score matching. The latter was used to generate a natural control group, persons who were not exposed to the *Pasos Adelante* program but otherwise empirically most similar to a matched group of survey respondents who did participate in the program. Of note, it was possible to identify four close matches, *i.e.*, the natural controls, for 77% of those in the survey who indicated they had participated in *Pasos*. 

The outcomes for the current study to the degree possible mapped those examined in previous shorter term evaluations [13,14,15] and those used in the Centers of Disease Control and Prevention’s Behavioral Risk Factor Surveillance System for identifying chronic disease health risk factors. The findings showed participants in *Pasos* were significantly more likely to meet CDC criteria for moderate or vigorous exercise in all three types of models tested. Additionally, whole milk consumption was lower in *Pasos* participants in the full sample (unadjusted and covariate adjusted) models. Though not significant in the propensity-score matched analysis, the magnitude of effect (OR = 0.29) was comparable to other models. There were no other significant differences among the other behavioral or clinical outcomes examined. The latter included body mass index (BMI), Waist-to-Hip ratio (WHR) and elevated glucose, measures that were positively impacted in evaluations based on following a higher number of program participants over a shorter period [15]. Overall, these findings provide further evidence for the effectiveness of *Pasos Adelante* in reducing chronic disease burden, and provide further support for the program in being identified as a research tested model for reducing chronic disease risk in Mexican origin Hispanics [16]. 

As *Pasos* is a community health worker/*promotora*-delivered chronic disease prevention program, the findings add to the growing consensus on the effectiveness and sustainability of community health worker-driven, community-based, prevention efforts with under-served populations [17,44,45,46]. Our study also complements all prior evaluations on this intervention by being the first to estimate effects with control or comparisons, and looking at longer term effects than prior studies. It should be noted however most participants were Mexican-origin women; similar to that observed with other diabetes management efforts using community health workers [47]. As diabetes is very high in Mexican origin men and Mexican origin male youth have the highest rates of obesity of the major subgroups in the US [48], novel chronic disease prevention models and programs focusing on recruiting and retaining Mexican origin men are warranted.

The findings also illustrated the utility of the propensity score matching approach to generate a natural group from community respondents matched to the survey’s *Pasos* participants. For this component of the study, all matched respondents were Hispanic (estimated 99% Mexican origin and around 80% were born in Mexico) and there were no significant differences in a range of covariates and potential confounders within the matched groups—yielding confidence to the causal interpretations. There has been a call for the increased use of propensity score matching for adjusting for selection biases in public health research, particularly that of epidemiological or clinical nature [26], for example it has been used to compare medical treatments using electronic health records [23]. However, this method has been infrequently applied for the evaluation of community health or health education interventions. This is despite its usefulness for generating evaluation results more similar to those of a randomized controlled trial, yet without community partner and individual participants’ burdens often associated with implementing those designs in community settings. Further, even if the community and partners agree to a randomized design evaluation of a community-based health promotion intervention, issues such as unequal groups (particularly with group randomization), low participation rates and treatment contamination, may hamper the strengths of the inferences from those studies in practice [49]. The current and related techniques should be used to complement and improve such efforts (e.g., the complications from unequal comparison groups in a community-based randomized trial can be reduced through the post-hoc application of propensity score methods).

## 5. Strengths and Limitations

There are notable strengths with the current study and approach. The outcomes examined were diverse, included behavioral and clinical measures that map prior intervention evaluation efforts well and are generally the standards for public health surveillance efforts. The random population-based survey was also conducted with outstanding response rate and was executed by trusted community health workers; this leads to additional confidence in the quality and accuracy of the data.

Two other particular strengths are the application of innovative evaluation methods that adjust for selection biases and the careful consideration of potential confounders. For both of the adjusted methods employed we used a range of plausible covariates and confounders that included demographics, acculturation factors, and additional factors. Those additional factors included potentially strong and diverse sources of selection biases, including diabetes status prior to the initiation of the initial *Pasos* study in the community, length of time residing in the targeted community, and beliefs around community empowerment to address diabetes. The latter psychosocial factor is a rarely considered type of confound in intervention evaluation research, yet there are likely such differences between persons that participate in a program such as *Pasos* and those that do not. Such differences may not be fully accounted for in the documented demographic and clinical conditions more often examined as confounding variables. 

The current study also has weaknesses. One is that the study was not powered for estimating effects of *Pasos Adelante* across its targeted outcome variables. This may be a source of discrepancies from prior evaluations of *Pasos* that had greater numbers of intervention participants and showed significant clinical effects, such effects were not observed in the current study. Also it is noted intervention exposure was determined by self-report. With the current data we do not have precise indicators on the time elapsed post-individuals’ completion, record of their individual treatment fidelity, nor account for whether the *Pasos* or non-*Pasos* exposed participants may have participated in other types of programs in the community. In addition, it is possible that respondents in the survey were not accurate in their recall of participation in *Pasos*. Providing confidence in our program exposure variable’s validity however, ancillary analysis suggests numbers of *Pasos* participants identified within the current sample is consistent with those projected from the proportional sampling of all adults in the community relative to the known number of *Pasos* participants [15] in the target community. 

It is also acknowledged that our determination of elevated glucose relied on primarily non-fasting samples. This test is not sufficient to clinically determine diabetes status, and the duration between participants’ last meal and the capillary could influence the measured glucose. That said, a rather conservative World Health Organization [35] standard of 200 mg/dL was used for non-fasting samples, and this was applied consistently across all comparison groups in the current study.

Another weakness is both adjustment techniques used rely on the identification of measured confounds that relate to intervention selection [26]. In both cases, applying regression based covariate adjustment and propensity score matching, we aimed to be as diverse as possible in accounting for potential clinical, behavioral, demographic and psychosocial differences in those that initially participated in *Pasos* versus those that do not. Despite those efforts there is always the possibility of unknown confounders. We did explore using an alternative technique to adjust for unmeasured confounders as well, adjusting effects through an instrumental variable analysis, however the potential instrumental variables considered did not meet the required assumptions of using that approach [26].

## 6. Conclusions

The current study adds to the evidence of the effectiveness of *Pasos Adelante* and related community-based chronic disease prevention interventions by showing significant and desired behavioral effects of participants’ examined 3–6 years post-completion of that intervention. This work also shows that the innovative use of secondary, community-representative data can complement other evaluations often limited in the inferences possible because of funding limitations or other practical considerations (e.g., the fact most research projects are funded 2–5 years, the higher costs of more complicated designs with multiple comparison groups and/or a control group; the ethical concerns and stakeholder reactivity to discussions of randomized trial designs within community settings). Programs such as *Pasos Adelante* that utilize community health workers are increasingly important to population-level efforts to stem the rising influence of chronic disease on mortality, quality of life and health care costs globally. 
